# Survey of Heteronormative Attitudes and Tolerance Toward Gender Non-conformity in Mountain West Undergraduate Students

**DOI:** 10.3389/fpsyg.2019.00793

**Published:** 2019-04-11

**Authors:** Steven G. Duncan, Gabrielle Aguilar, Cole G. Jensen, Brianna M. Magnusson

**Affiliations:** ^1^Department of Microbiology and Molecular Biology, College of Life Sciences, Brigham Young University, Provo, UT, United States; ^2^Department of Public Health, College of Life Sciences, Brigham Young University, Provo, UT, United States

**Keywords:** heteronormativity, attitudes, gender non-conformity, sex roles, tolerance, discrimination, social norms, heterosexism

## Abstract

**Introduction:**

Heteronormative attitudes are prevalent in the United States and may contribute to discrimination against individuals who do not conform to traditional gender roles. Understanding the attitudes of undergraduate students is of particular interest as they may represent emergent societal views toward gender non-conformity.

**Materials and Methods:**

We conducted an online survey of Mountain West college students between the ages of 18–24 years to assess perceptions of personal gender conformity using the Traditional Masculinity-Femininity Scale (TMF), endorsement of heteronormative beliefs using the Heteronormative Attitudes and Beliefs Scale (HABS), and explicit tolerance of gender non-conformity on a seven-point Likert Scale.

**Results:**

The sample (*n* = 502) was 84% female and 78% white. Approximately 21% of respondents identified as a sexual minority and 36% identified as liberal or somewhat liberal (27% were conservative). The mean score on the TMF was 5.23 (95% CI: 5.15–5.32), indicating moderate levels of personal gender conformity. The mean HABS score was 3.31 (95% CI: 3.19–3.43), indicating relatively low endorsement of heteronormative attitudes. TMF and HABS scores were both highest in heterosexual males. Most respondents (73%) were taught traditional gender roles in their childhood home, and 89% had heard negative opinions about non-conformity. The majority (80.6%) of respondents reported that they know someone who displays non-conforming characteristics and 61% said that they associate gender non-conformity with homosexuality. Approximately, 7% reported they had bullied others for not conforming to their gender. Among heterosexuals, 13.6% reported they had been bullied for gender non-conformity as did 42.7% of LGBTQ-identified individuals. Nearly 1-in-4 (23.6%) believed that male cross-dressing is wrong. Nearly 1-in-5 (17.2%) agreed with the statement that those who dress or act like the opposite sex were more likely to be abused or neglected during their development.

**Conclusion:**

Students reported relatively low endorsement of heteronormative attitudes and moderate levels of acceptance toward gender non-conforming persons. The sample may reflect shifting attitudes when compared with outside data sets.

## Introduction

Social role theory posits that male and female genders have different roles resulting from biological, ecological, and social differences (Eagly, 1987; Ellemers, 2018; Huang et al., 2018). These specialized roles have shaped our societal conception of masculinity and femininity over time. Behaviors that match stereotypical traits and roles of one’s biological sex are gender conforming and tend to be reinforced, while behaviors that do not match the traits and roles stereotypically assigned to one’s biological sex are gender non-conforming and often discouraged (Thomas and Blakemore, 2013; Spivey et al., 2018). Gender non-conformity is “the extent to which individuals fail to conform to gender-based societal proscriptions concerning appearance, feelings, or behaviors” (Martin-Storey and August, 2016). This includes gender incongruent behavior related to interests, occupation, and traditional gender characteristics within marital relationships or domestic partnerships (Cook et al., 2013).

Negative attitudes toward gender non-conformity are pervasive in American culture (Friedman and Downey, 1999; Skidmore et al., 2006; Schwartz et al., 2016), and contribute to much of the violence and discrimination commonly experienced among sexual minorities (Balsam et al., 2005; Skidmore et al., 2006; Rosa et al., 2018) and those who do not conform to traditional gender norms (Fasoli et al., 2017). This may be due in part to the widespread acceptance of heteronormative attitudes in the United States (Herek, 2009; Baams et al., 2015).

Heteronormativity is narrowly defined as preferential attitudes toward heterosexual relationships. However, heteronormative attitudes are strongly rooted in the idea that males and females have distinct characteristics and roles which are accompanied by fixed behavioral expectations for males and females. The endorsement of heteronormative attitudes may be associated with negative attitudes toward gender non-conformity (Habarth, 2015). The possibility of a correlation is worthy of consideration as gender non-conforming individuals may experience physical and social harm as a result of this bias.

Evidence suggests that heteronormative bias and heterosexism are disseminated through cultural institutions, leading to prevalent bias against sexual minorities (Skidmore et al., 2006; Math and Seshadri, 2013). Early attitudes about gender roles and expectations of conformity are shaped to some extent by parents’ modeled behavior (Halpern and Perry-Jenkins, 2016). It has been posited that negative attitudes may stem from the perception of gender non-conforming behavior as a threat to society’s traditional sex role structure (Whitley, 1987; Rosa et al., 2018), suggesting higher personal gender conformity likely correlates with a negative view of non-conformity. If underlying heteronormative ideologies may be associated with harmful reactionary behavior, this association should be carefully studied and reported.

There is abundant empirical evidence demonstrating that the violation of gender roles is strongly associated with negative reactions from others. Individuals who violate gender roles often face prejudice and discrimination in social and employment situations (Friedman and Downey, 1999; Li et al., 2016). These reactions can be more pronounced toward males exhibiting gender non-conformity as stereotypes of male traits and roles are relatively less flexible than female stereotypes (Diekman et al., 2004). However, in some cases atypical men may be defaulted the “benefit of the doubt” as their status in patriarchal society is higher (Moss-Racusin et al., 2010). Anyone not conforming to gender roles can become the target of discrimination regardless of sexual orientation.

Living as a gender non-conforming individual has been associated with a higher risk of enduring social stressors including stigmatization and poorer health-related quality of life outcomes (Gordon et al., 2017). In a recent study conducted in Massachusetts, 65% of gender non-conforming individuals reported public accommodations discrimination in the past 12 months, especially in transportation, retail, and healthcare (Reisner et al., 2015). Internalized negative attitudes toward gender non-conformity among LGBT individuals who exhibit atypical sex role characteristics are associated with psychological distress, feelings of inadequacy, and mental health problems (Bailey et al., 1997; Skidmore et al., 2006; Schwartz et al., 2016; Hart et al., 2018). In turn, perceived discrimination positively correlates with greater psychiatric morbidity risk including suicidal ideations (Mays and Cochran, 2001; Sutter and Perrin, 2016), an association well-documented in populations when gender non-conformity increases an individual’s visibility as a target for discrimination and social ostracization (Skidmore et al., 2006; Martin-Storey and August, 2016).

Additionally, heteronormative attitudes can negatively affect individuals who are traditionally gender conforming. Gendered norms reflect differences in the value placed on girls versus boys, and this disparity leads to lower self-esteem in individuals who dress and behave according to feminine norms (Sen and Ostlin, 2007; Bhattacharya and Shukla, 2014). Men who experience conflict over violating the male gender role by expressing emotion or displaying affection toward other men are less likely to seek professional psychological help for mental illness and tend to have more negative attitudes toward help-seeking in general (Good et al., 1989; Davis and Liang, 2015). Men have also been found to encounter backlash when engaging in female-dominated communal roles (Croft et al., 2015). While these examples are not comprehensive and there are positive aspects of adhering to traditional norms, it is clear that heteronormative reinforcement of gender roles affects everyone.

College students constitute an important population for researching gender norms as they fall in a life stage characterized by the rapid development of personal values and identity (Potts, 2017). They also represent a more diverse group of students than the subset that pursues graduate education. Surveying an undergraduate population is particularly important for this study as discrimination against LGBT students, many of whom are not gender conforming, commonly occurs on college campuses (Seelman et al., 2017). There is currently no published literature characterizing attitudes toward gender non-conformity or describing its associated correlates within a population of young adults.

A survey of undergraduate students may represent emergent attitudes of an influential new population, and responses may indicate the future direction of general tolerance toward gender non-conformity and an expected trajectory of gender roles in American society. This knowledge will be critical to understanding and navigating societal expectations related to gender. Designing and implementing interventional measures to combat discriminatory attitudes must begin with an in-depth understanding of tolerance within a population and the key factors that contribute to its development.

The purpose of this study was to examine attitudes toward gender non-conformity in conjunction with heteronormative beliefs and the respondent’s own degree of gender conformity in a population of undergraduate students throughout the Mountain West. Data were collected with the purpose of elucidating how attitudes toward gender norms may correlate with intolerant and potentially discriminatory views and behaviors. This knowledge could foster tolerance and sensitivity toward gender non-conforming individuals and associated minority groups. Additionally, the collected data describe the relationship between heterosexism, gender conformity, and tolerance using validated survey instruments which previously have not been correlated. These associations provide valuable new insight into the complex determinants that drive production and propagation of gender reinforcement.

## Hypotheses

We hypothesized that there would be a positive association between endorsement of heteronormative attitudes and personal gender conformity and that both heteronormative attitudes and gender conformity would differ by characteristics including biological sex and sexual orientation. We further hypothesized that heteronormative attitudes and personal gender conformity would correlate with tolerance toward gender non-conforming individuals.

## Materials and Methods

A cross-sectional internet panel survey of 502 United States college aged adults in the Mountain West was conducted in 2017. Participants were recruited through Qualtrics (Provo, UT, United States), a worldwide software company specializing in market research. Qualtrics operates an actively managed, global proprietary panel of registered members and recruits through social media, web publishers and global partners. Because the study was implemented online without recruiters or interviewers, no personnel training was necessary.

Panelists who met initial screening criteria (age and state of residence) received an email invitation with a link to the survey, as well as information regarding the approximate length of the survey and quantity of reward points (a value of US$ <2.00) that would be credited to their account upon completion. The survey link brought potential participants to an informed consent page. After consenting to participate, respondents were screened using two additional questions to ensure they met all eligibility criteria (18–24 years of age and college enrollment). The study was approved by the [Brigham Young University] Institutional Review Board.

### Measurement

The survey consisted of 56 items and was comprised of 10 demographic questions, The Heteronormative Attitudes and Beliefs Survey (HABS), The Traditional Masculinity-Femininity Scale (TMF), and 24 additional questions assessing explicit tolerance. Details on these measures follow.

### Demographics

Nine demographic questions, taken from the 2014 General Social Survey (Smith et al., 2015) were included. Racial categories were slightly modified to satisfy IRB requirements and improve clarity. Respondents reported age in years, race, biological sex, marital status, sexual orientation (heterosexual or sexual minority), level of education, perceived social class, political affiliation, religious affiliation, and religiosity.

### Heteronormative Attitudes and Beliefs Scale (HABS)

The 16-item HABS was used to measure endorsement of heteronormative attitudes (Habarth, 2015). Respondent’s indicated agreement on a seven-point Likert scale for each item. To calculate overall HABS scores, negatively worded items (#1, 4, 9, 11, 12, 15, 16) were reverse coded and all items were averaged to create a score ranging from 1 to 7, with higher values indicating greater endorsement of heteronormative beliefs. The HABS can be divided into two subscales: the Essential Sex and Gender subscale (Items: #3, 5, 6, 7, 9, 11, 12, 15) which addresses beliefs about gender, and the Normative Behavior subscale (items: #1, 2, 4, 8, 10, 13, 14, 16) which addresses sex role expectations within romantic relationships. Subscale scores were created by averaging relevant items.

### Traditional Masculinity-Femininity Scale (TMF)

The TMF measured respondents’ personal gender conformity (Kachel et al., 2016). The scale is comprised of six statements related to self-perception, interests, appearance, attitudes, and beliefs regarding which participants rank themselves from “Totally Masculine” to “Totally Feminine” on a seven-point scale. The score on these six items is averaged to create an overall value for TMF. Scoring of the TMF is dependent on the respondent’s self-reported biological sex such that male respondents’ higher scores on the TMF correspond to higher self-perceived masculinity and female respondents’ higher scores on the TMF correspond to higher self-perceived femininity.

### Explicit Tolerance

Explicit tolerance toward individuals with atypical sex role characteristics was assessed using a total of 24 questions primarily developed by the authors. Respondents were asked to indicate agreement with the following 13 statements (on a seven-point Likert scale): “My males friends are masculine,” “Men who are not masculine are good role models,” “I think less of men who have feminine mannerisms,” “Men should not act like women in the workplace,” “I prefer men to be feminine rather than masculine,” “My female friends are feminine,” “Women who are not feminine are good role models,” “I think less of women who have masculine mannerisms,” “Women should not act like men in the workplace,” “I prefer women to be masculine rather than feminine,” “My friends are accepting of people who do not conform to traditional gender roles,” “I feel restricted by the gender label that people attach to me,” and “I feel restricted by the expectations people have of me because of my gender.” The final two questions in this list were from the 7-factor measure of sexual prejudice developed by Dr. Sean G. Massey, each about a tendency to resist heteronormative expectations (Massey, 2009).

The remaining eleven items simply asked respondents to indicate whether they agreed or disagreed with each statement. Possible factors that may influence the development of certain gendered attitudes were addressed (i.e., upbringing, associations). Participants were asked to report how tolerant they perceive their friends and peers to be, as well as whether or not they have witnessed discrimination toward gender non-conforming individuals. Four of these questions were from the open-source 2015 South African Social Attitudes Survey to assess self-reported personal conformity, level of tolerance, and past involvement with bullying related to non-conformity (Roberts et al., 2016). These questions were slightly modified to better fit our target demographic, and several were split into multiple questions for clarity.

### Statistical Analysis

Frequencies and proportions were calculated to summarize categorical variables and means and 95% confidence intervals were used for continuous variables. Bivariate associations by sex and sexual orientation were evaluated using a chi-square test for categorical variables and a *t*-test for difference in means for continuous variables.

## Results

A total of 511 people began the survey and 502 completed it. Table 1 shows the demographic characteristics of respondents. The majority of participants were female (83.9%), heterosexual (78.9%) and white, non-hispanic (77.7%). We examined the distribution of demographic characteristics by biological sex and sexual orientation. In general, males and females were not different with respect to demographic variables. One notable exception is that a higher percentage of female respondents (23.3% vs. 8.6%) identified as being “gay, lesbian, or bisexual.” Similarly, the distribution of demographic characteristics by sexual orientation shows that respondents who identified as gay, lesbian, or bisexual reported a higher percentage of liberal or slightly liberal political affiliation (66.7% vs. 27.7%) when compared with heterosexual respondents.

**Table 1:** Demographic characteristics of a sample of (*n* = 502) college-aged students in the Mountain-West.

	Total sample *n* (%)	Male *n* (%)	Female *n* (%)	*p*-value^a^	Heterosexual *n* (%)	Sexual minority *n* (%)	*p*-value^a^
Age in years (mean, SD)	20.45 (1.87)	20.65 (2.01)	20.42 (1.84)		20.49 (1.85)	20.30 (1.90)	
Sex							
Male	81 (16.1)	–	–		74 (18.6)	7 (6.7)	^∗∗^
Female	421 (83.9)	–	–		323 (81.4)	98 (93.3)	
Sexual orientation							
Heterosexual	397 (78.9)	74 (91.4)	323 (76.7)	^∗∗^	–	–	
Gay, lesbian, or bisexual	106 (21.1)	7 (8.6)	98 (23.3)		–	–	
Race/ethnicity							
White, non-hispanic	391 (77.7)	61 (75.3)	330 (78.4)		310 (78.1)	81 (76.4)	
Other race or ethnicity	112 (22.3)	20 (24.7)	91 (21.6)		87 (21.9)	25 (23.6)	
Marital status							
Never married	426 (85.2)	73 (90.1)	353 (84.3)		333 (84.3)	91 (88.4)	
Married	68 (13.6)	8 (9.9)	60 (14.3)		58 (14.7)	10 (9.7)	
Divorced	6 (1.2)	0 (0.0)	6 (1.4)		4 (1.0)	Suppressed^b^	
Social class							
Lower/working class	211 (42.0)	32 (39.5)	179 (42.5)		165 (41.6)	46 (43.8)	
Middle/upper class	271 (54.0)	47 (58.0)	224 (53.2)		218 (54.9)	53 (50.5)	
Don’t know	20 (4.0)	Suppressed^b^	18 (4.3)		14 (3.5)	6 (5.7)	
Political views							
Liberal	107 (21.3)	14 (17.3)	93 (22.1)		59 (14.9)	48 (45.7)	^∗∗∗^
Slightly liberal	73 (14.5)	7 (8.6)	66 (15.7)		51 (12.9)	22 (21.0)	
Moderate	108 (21.5)	21 (26.0)	87 (20.7)		87 (21.9)	21 (20.0)	
Slightly conservative	74 (14.7)	13 (16.1)	61 (14.5)		73 (18.4)	Suppressed^b^	
Conservative	61 (12.2)	15 (18.5)	46 (10.9)		59 (14.9)	Suppressed^b^	
Don’t know	79 (15.7)	11 (13.6)	68 (16.2)		68 (17.1)	11 (10.5)	
Religious affiliation							
Christian	302 (61.4)	52 (65.0)	250 (60.7)		270 (69.4)	32 (31.1)	^∗∗∗^
No religion	165 (33.5)	26 (32.5)	139 (33.7)		104 (26.7)	61 (59.2)	
Something else	25 (5.1)	Suppressed^b^	23 (5.6)		15 (3.9)	10 (9.7)	


*^a^p-values from a chi-square tests for the difference of proportions. ^*b*^Cells with five or fewer responses were suppressed. ^∗∗^*p* < 0.01, ^∗∗∗^*p* < 0.001.*

The average TMF score for all respondents was 5.23 (95% CI: 5.14–5.31) and the average HABS score was 3.42 (95% CI: 3.35–3.59). Table 2 gives the TMF and HABS scores for the total population and separated by sex and sexual orientation. For both the TMF and the HABS scales, heterosexuals averaged higher scores than did females and those identifying with a sexual minority.

**Table 2:** Mean scores on the Traditional Masculinity and Femininity Scale and Heteronormative Attitudes and Beliefs for the total population and by biological sex and sexual orientation.

	Total population (*n* = 502)	Males (*n* = 81)	Females (*n* = 421)	Heterosexual (*n* = 397)	Sexual minority (*n* = 105)
			
	Mean (95% CI)	Mean (95% CI)		Mean (95% CI)	
Traditional Masculinity and Femininity Scale^a^	5.23 (5.14–5.31)	5.70 (5.49–5.92)	5.14 (5.05–5.23)	5.37 (5.28–5.46)	4.68 (4.51–4.86)
Heteronormative Attitudes and Beliefs Scale^b^	3.42 (3.35–3.59)	4.15 (3.92–4.38)	3.34 (3.21–3.48)	3.80 (3.67–3.93)	2.24 (2.06–2.41)
Normative Behavior Subscale^b^	3.16 (3.04–3.28)	3.67 (3.42–3.92)	3.06 (2.93–3.20)	3.47 (3.33–3.60)	2.01 (1.84–2.18)
Essential Sex and Gender Subscale^b^	3.96 (3.82–4.11)	4.85 (4.56–5.13)	3.80 (3.64–3.95)	4.33 (4.18–4.48)	2.59 (2.34–2.83)


*^a^Traditional Masculinity and Femininity Scale (TMF) is comprised of six statements ranked on a seven-point Likert scale from Totally Masculine to Totally Feminine. Scoring of the TMF is dependent on the respondent’s biological sex such that higher scores indicated higher conformity with one’s biological sex. ^*b*^Heternormative Attitudes and Beliefs Scale is comprised of 16 items divided into two subscales (Normative Behavior Subscale and Essential Sex and Gender Subscale) ranked on a seven-point Likert scale. Higher values indicate greater endorsement of heteronormative beliefs.*

Table 3 presents data on agreement with six statements consistent with heteronormative attitudes and beliefs and seven statement inconsistent with heteronormative attitudes and beliefs. The majority of persons in the sample agreed with the statements “My male friends are masculine (72.1%)” and “my female friends are feminine (81.1%).” Few respondents agreed with the statements, “I think less of men who have feminine mannerisms (14.0%)” and “I think less of women who have masculine mannerisms (7.8%).” Consistent with other results, a smaller percentage of females and those identifying with a sexual minority agreed with statement representing heteronormative attitudes or beliefs. Conversely, most (77.1%) respondents agreed with the statement, “My friends are accepting of people who do not conform to traditional gender roles.” A larger percentage of females and those identifying with a sexual minority agreed with this and other statements inconsistent with heteronormative attitudes and beliefs.

**Table 3:** Agreement with statements that are consistent and inconsistent with heteronormative attitudes and beliefs in the total sample and by biological sex and sexual orientation in a sample of college students in the Mountain-West.

	Strongly agree, somewhat agree, or agree						
	Total sample	Biological sex			Sexual orientation		
	*n* (%)	Male *n* (%)	Female *n* (%)		Heterosexual *n* (%)	Sexual minority *n* (%)	
**Statements consistent with heteronormative attitudes**^a^							
My male friends are masculine	361 (72.1)	65 (80.3)	296 (70.5)		304 (76.8)	57 (54.3)	^∗∗∗^
My female friends are feminine	407 (81.1)	66 (80.5)	341 (81.0)		338 (85.1)	69 (65.7)	^∗∗∗^
I think less of men who have feminine mannerisms	70 (14.0)	18 (22.2)	52 (12.4)	^∗^	68 (17.2)	2 (1.9)	^∗∗∗^
I think less of women who have masculine mannerisms	39 (7.8)	14 (17.3)	25 (5.9)	^∗∗∗^	39 (9.8)	0	^∗∗∗^
Men should not act like women in the work place	119 (23.7)	31 (38.3)	88 (20.9)	^∗∗∗^	144 (28.7)	5 (4.8)	^∗∗∗^
Women should not act like men in the work place	81 (16.1)	23 (28.4)	58 (13.6)	^∗∗^	78 (19.7)	3 (2.9)	^∗∗∗^
							
**Statements not consistent with heteronormative attitudes**^a^							
Men who are not masculine are good role models	202 (40.2)	23 (28.4)	179 (42.5)	^∗^	149 (37.5)	53 (50.5)	^∗^
Women who are not feminine are good role models	195 (38.8)	24 (29.6)	171 (40.6)		145 (36.5)	50 (47.6)	^∗^
I prefer men to be feminine rather than masculine	35 (7.0)	9 (11.1)	37 (8.8)		30 (7.6)	16 (15.2)	^∗^
I prefer women to be masculine rather than feminine	46 (9.2)	7 (8.6)	28 (6.7)		24 (6.1)	11 (10.5)	
My friends are accepting of people who do not conform to traditional gender roles	387 (77.1)	51 (63.0)	336 (79.8)	^∗∗^	290 (73.1)	97 (92.4)	^∗∗∗^
I feel restricted by the gender labels that people attach to me	79 (15.7)	8 (9.9)	71 (16.9)		42 (10.6)	37 (35.2)	^∗∗∗^
I feel restricted by the expectations that people have of me because of my gender	188 (37.5)	11 (13.6)	177 (42.0)	^∗∗∗^	144 (28.7)	74 (70.5)	^∗∗∗^


*^a^Questions were evaluated on a seven-point Likert scale from strongly disagree to strongly agree. ^∗^*p* < 0.05, ^∗∗^*p* < 0.01, ^∗∗∗^*p* < 0.001.*

Tables 4, 5 present information on 11 questions assessing explicit tolerance. Table 4 provides the percentage of respondents who agreed with each statement. Statements receiving the highest percentage endorsement include, “I have heard negative opinions about people who dress or act like the opposite sex” which was endorsed by 89.4% of the sample and “I personally know someone who does not conform to their gender” (80.6%). A majority (60.7%) of respondents indicated that they associate dressing or acting like the opposite sex with homosexuality. Nearly 1-in-5 (17.2%) agreed with the statement that those who dress or act like the opposite sex were more likely to be abused or neglected during their development. The HABS and TMF scores varied across tolerance statements as would be expected. Higher HABS scores (stronger endorsement of heteronormative attitudes and beliefs) and TMF (stronger endorsement of personal gender conformity) were associated with agreement with statements that expressed less tolerance.

**Table 4:** Agreement with statements assessing explicit tolerance in the total sample and by biological sex and sexual orientation in a sample of college students in the Mountain-West^a^.

	Strongly agree, somewhat agree, or agree						
	Total sample *n* (%)	Male *n* (%)	Female *n* (%)	*p*-Value	Heterosexual *n* (%)	Sexual minority *n* (%)	*p*-Value
In my childhood home, I was taught that men should act like men and women should act like women	366 (73.1)	63 (78.8)	303 (72.0)		297 (75.0)	69 (65.7)	
I associate people who dress or act like the opposite sex with homosexual tendencies	304 (60.7)	52 (65.0)	252 (59.9)		252 (63.6)	52 (49.5)	^∗∗^
I personally know someone who does not conform to their gender	404 (80.6)	54 (67.5)	350 (83.1)	^∗∗^	307 (77.5)	97 (92.4)	^∗∗∗^
If someone who dresses or acts like the opposite sex, it is more likely they were abused or neglected during development	86 (17.2)	19 (23.8)	67 (16.0)		77 (19.4)	9 (8.7)	^∗∗^
I have heard negative opinions about people who dress or act like the opposite sex	448 (89.4)	64 (80.0)	384 (91.2)	^∗∗^	349 (88.1)	99 (94.3)	
I think it is wrong when men dress like women	119 (23.8)	28 (35.0)	91 (21.6)	^∗^	117 (29.6)	Suppress	^∗∗∗^
I think it is wrong when women dress like men	89 (17.8)	20 (25.0)	69 (16.4)		88 (22.2)	Suppress	^∗∗∗^
I think it is entirely natural for some men to dress and act like women	339 (67.7)	44 (55.0)	295 (70.1)	^∗^	240 (60.6)	99 (94.3)	^∗∗∗^
I have witnessed teasing or bullying of people who dressed or acted like someone of the opposite sex	369 (73.7)	49 (61.3)	320 (76.0)	^∗∗^	278 (70.2)	91 (86.7)	^∗∗∗^
In the past, I have teased or bullied someone who dressed or acted like the opposite sex	35 (7.0)	12 (15.0)	23 (5.5)	^∗∗^	31 (7.8)	4 (3.8)	
In the past, I have been teased or bullied for acting like the opposite sex	99 (19.8)	10 (12.5)	89 (21.1)		54 (13.6)	45 (42.9)	^∗∗∗^


*^a^Questions were evaluated on a seven-point Likert scale from strongly disagree to strongly agree. ^∗^*p* < 0.05, ^∗∗^*p* < 0.01, ^∗∗∗^*p* < 0.001.*

**Table 5:** Average Heteronormative Attitudes and Beliefs Scale score (HABS) and Traditional Masculinity and Femininity Scale score (TMF) for those who agreed and disagreed with each statement assessing explicit tolerance.

Explicit tolerance questions	Heteronormative Attitudes and Beliefs Scale			Traditional Masculinity and Femininity Scale		
		
	Agree	Disagree		Agree	Disagree	
				
	Mean (95% CI)		*p*-value^a^	Mean (95% CI)		*p*-value^a^
I think it is wrong when men dress like women	5.13 (4.96–5.31)	2.96 (2.85–3.07)	^∗∗∗^	5.66 (5.47–5.85)	5.11 (5.01–5.20)	^∗∗∗^
I think it is wrong when women dress like men	5.20 (5.10–5.46)	3.08 (2.97–3.19)	^∗∗∗^	5.77 (5.55–5.98)	5.12 (5.03–5.21)	^∗∗∗^
I think it is entirely natural for some men to dress and act like women	2.83 (2.72–2.94)	4.82 (4.66–4.99)	^∗∗∗^	5.07 (4.97–5.17)	5.58 (5.43–5.73)	^∗∗∗^
In the past, I have teased or bullied someone who dressed or acted like the opposite sex	3.76 (3.35–4.17)	3.45 (3.33–3.58)		5.16 (7.79–5.54)	5.24 (5.15–5.33)	
In my childhood home, I was taught that men should act like men and women should act like women	3.68 (3.53–3.83)	2.92 (2.74–3.11)	^∗∗∗^	5.27 (5.17–5.37)	5.14 (4.98–5.31)	
If someone who dresses or acts like the opposite sex, it is more likely they were abused or neglected during development	4.44 (4.18–4.69)	3.28 (3.15–3.41)	^∗∗∗^	5.32 (5.07–5.56)	5.22 (5.13–5.31)	
I associate people who dress or act like the opposite sex with homosexual tendencies	3.83 (3.68–3.98)	2.93 (2.75–3.11)	^∗∗∗^	5.34 (5.24–5.45)	5.07 (4.94–5.21)	^∗∗^
I personally know someone who does not conform to their gender	3.36 (3.22–3.50)	3.97 (3.72–4.21)	^∗∗∗^	5.17 (5.08–5.26)	5.51 (5.30–5.72)	^∗∗^
I have heard negative opinions about people who dress or act like the opposite sex	3.45 (3.33–3.59)	3.61 (3.27–3.95)		5.23 (5.14–5.32)	5.31 (5.01–5.60)	
I have witnessed teasing or bullying of people who dressed or acted like someone of the opposite sex	3.30 (3.15–3.43)	3.99 (3.75–4.24)	^∗∗∗^	5.18 (5.08–5.28)	5.38 (5.20–5.56)	^∗^
In the past, I have been teased or bullied for acting like the opposite sex	2.75 (2.51–3.00)	3.65 (3.52–3.79)	^∗∗∗^	4.56 (4.37–4.75)	5.40 (5.31–5.49)	^∗∗∗^


*^a^p-values are derived from t-tests. ^∗∗∗^p < 0.05, ^∗∗^p < 0.01, ^∗∗^p < 0.001.*

## Discussion

### General Observations

Based upon averaged responses to the HABS, TMF, and explicit tolerance questions, students in the sample reported moderate tolerance toward gender non-conformity, moderately high personal conformity, and relatively low endorsement of heteronormative attitudes. Correlations show support for the authors’ hypotheses, indicating there is a relationship between all three variables: gendered self-expression, acceptance of traditional attitudes, and tolerance toward gender non-conformity.

The strength of the correlation between HABS, TMF, and tolerance scores was offset by respondents who did not fall into the two principal clusters: conforming, conventional thinkers with lower tolerance and non-conforming, unconventional thinkers with higher tolerance. Some of these confounding students were both highly tolerant of non-conformity and simultaneously highly conforming themselves. A few were intolerant of their own lower personal conformity, and they perhaps experience some measure of cognitive dissonance or internalized feelings of negativity (Hart et al., 2018). It appears that individual attitudes about conformity and gender are interrelated but do not always align perfectly with the hypothesized model.

### Comparing Populations

Our sample was largely female and white, non-hispanic. It is unclear whether females were more likely to be invited to participate in the survey or more likely to respond to an invitation. Regardless, our sample population differs in important ways from the demographic characteristics of the general population of college students in the United States. Specifically, related to biological sex and sexual orientation. Our sample contained a larger proportion of LGBTQ-identified respondents than the national average: 21% compared to 7.9% of individuals aged 18–29 as provided by the 2014 GSS or the Gallup poll’s estimated 8.1% among millennials (Newport, 2018). Both females and those who identify with a sexual minority are likely to feel that a survey about gender issues is more applicable to them, increasing response rates in these populations. Additionally, our results suggest that females and those identifying as lesbian, gay, or bisexual have lower endorsement of heteronormative attitudes. As such, our data likely do not represent the population of United States college students generally.

In the full sample, the mean score on the HABS was 3.42; 3.16 on the normative behavior subscale and 3.96 on the essential sex and gender subscale. A small (*n* = 84) 2015 study of undergraduate psychology students used to validate the HABS, identified scores of 2.41 on the normative behavior and 3.08 on the essential sex and gender subscale (Habarth, 2015). Similar to our sample, the psychology student volunteers were 76% female, but were more diverse in terms of racial and ethnic minority. Our sample’s higher endorsement of heteronormative attitudes may reflect differences in the general beliefs between students in the Mountain West, a traditionally conservative enclave, and those at the University of Michigan where the first study took place. Additionally, approximately half the students in the validation study were recruited from a gender and sexual identities-focused course which may have attracted students who already had lower heteronormative beliefs or possibly, converted students to be more open-mined with respect to gender and sexual identities.

A second 2017 study of social workers in the United Kingdom (UK) (*n* = 112) also identified significantly lower endorsement of heteronormative attitudes than observed in our sample (Schaub et al., 2017). The HABS scores, converted to averages, were 2.09 for the total HABS, and 1.93 for the normative beliefs subscale and 2.27 for the essential sex and gender subscale. The United Kingdom sample, like ours, was predominately female (75%), but was significantly more diverse with respect to other demographic characteristics, including approximately 35% of respondents who identified as sexual minorities. Additionally, those who pursue social work as a profession are likely to be more accepting of all persons than the general population.

In our sample, responses on the TMF showed that men were somewhat more gender conforming on average (5.7) than women (5.14). Validation studies for the TMF from German universities (Kachel et al., 2016) identified a mean score of 5.28 in females, similar to the score in our sample. Conversely, the same study identified a TMF score for men of 4.44, significantly lower than we observed. The difference in self-reported gender conformity for men may reflect differences in gender expectations for men in Europe versus the United States.

With respect to explicit tolerance, our sample was generally more tolerant than previous samples. According to the 2015 South African Social Attitudes Survey (SASAS) reported (Roberts et al., 2016), 66.5% of 3,115 respondents agreed that cross-dressing is disgusting. We observed a much lower proportion of respondents endorsing this belief, specifically, 23.8% and 17.7% of our sample agreed cross-dressing was wrong for men and women, respectively. Similarly, the SASAS reported that 32.6% of respondents felt it was entirely natural for some men to want to dress and act feminine while we observed 67.7% endorsement for this statement. The SASAS reported that 6.9% admitted to shouting or teasing someone because of their gender non-conformity, consistent with 7% reporting in our sample. The SASAS sample surveys all ages of the South African population. The relatively tolerant attitudes observed in our sample may reflect the age differences in the population or differences in gender attitudes between South Africa and the United States.

### Increasing Numbers of LGBT-Identified Youth

The demographic breakdown of survey respondents showed more sexual minorities among female respondents. This may indicate more openness in identifying as a sexual minority for females than males, perhaps due to increasing societal tolerance of sexual exploration in females. Female sex roles tend to be less stringently reinforced with the advent of modern feminism (Diekman et al., 2004), which may contribute to more flexibility in reporting sexual orientation when LGBTQ identity intersects with gender identity. The Gallup poll shows a sharp increase in individuals between the ages of 19 and 38 identifying as a sexual minority – from 5.2 to 8.1% in the last 6 years (Newport, 2018).

The gender gap between women and men identifying as LGBTQ has expanded from 0.1 to 1.2% across all ages (Newport, 2018), with self-identifying LGBTQ women slightly outnumbering men. This gap was even larger in the data collected for this survey (14.7% more LGBTQ women than men). Regardless of any proposed rationale for this, LGBTQ undergraduate students in the Mountain West seem to mirror a larger trend throughout the United States of increasing self-acceptance or endorsement of sexual fluidity, particularly in females.

### Gender Non-conformity and LGBT Identity

There is evidence that gender non-conforming children are likely to be subjected to assumptions of deviant sexual orientation, especially in the case of strongly feminine boys (Thomas and Blakemore, 2013). Gender non-conformity has been previously associated with psychological distress in gay men (Skidmore et al., 2006). This may stem from a stricter enforcement of masculine roles and a patriarchal society’s preference for masculine traits.

Historically, gender non-conformity and sexual non-conformity have not necessarily been linked concepts (Bullough, 2008; Hart et al., 2018). A recent study showed that discriminatory attitudes surfaced when listeners heard gender-atypical vocal cues. Intolerance tended to intensify when individuals reported assuming a non-heterosexual orientation in the speaker (Fasoli et al., 2017), indicating that sexual orientation can compound intolerance but is not necessary to provoke it. Anyone found to be out of conformity with traditional gender roles can become the target of discrimination.

The association between gender non-conformity and homosexuality was further expounded by participants in this survey; approximately 61% reported making the association. This result suggests that peer-perceived gender non-conformity is accompanied by presupposed homosexuality in the majority of cases: a claim which agrees with other recent research and may depend upon judgment criteria (Fasoli et al., 2017). The link between gender non-conformity is pronounced enough that sexual minorities sometimes report they were bullied for their orientation when they were actually mistreated for their gender non-conformity (Bos and Sandfort, 2015).

Approximately 17% of respondents reported an association between gender non-conformity and suspected neglect/abuse during development. Current literature indicates that gender non-conformity is a risk indicator for childhood physical, sexual, and psychological abuse, independent of sexual orientation (Roberts et al., 2012). Additionally, increased physical and emotional abuse have been reported in non-heterosexuals, with gender atypicality as a possible causal factor (Corliss et al., 2002; Rosa et al., 2018). Adult femininity has been associated with childhood sexual abuse in gay and bisexual men (Sandfort et al., 2007). Although the assumption that early abuse or neglect necessarily gave rise to non-conforming behavior may be baseless, the association between gender non-conformity and childhood abuse is rooted in reality. In future studies, it would be important to separate these ideas into separate questions in order to distinguish assumed causation from compassionate understanding.

### Peer Perception and Self-Identification

The perception of gender conformity in others may differ from self-reported gender conformity. Kite and Deaux (1987) noted that people often subscribe to an ‘implicit inversion theory’ in which homosexuals are assumed to be similar to heterosexuals of the opposite sex; this assumption stems from belief in a binary model of human gender expression.

While this theory can hold true when perceiving others, homosexuals tend to self-stereotype and most often consider themselves in possession of cisgender or sex-typical characteristics. For example, gay men tend to report higher masculinity and lower femininity than lesbian women on the TMF (Kachel et al., 2016). These two conflicting systems of perception present an interesting dichotomy: openly gay individuals may perceive themselves to be more gender conforming (via self-stereotyping) than peers who prescribe to inversion theory. Because the survey requested self-evaluation of masculinity and femininity on the TMF, data may be incomplete. A future study might include an additional measure of participants’ gender conformity from a fixed outside perspective.

### Response Bias

Self-selection and non-response bias among participants who take opt-in surveys has led to unbalanced gender representation in other studies (Hill and Shaw, 2013). In North America, multiple surveys of the general population have observed single, childless males to be prototypical non-responders (Ness et al., 2010; Rueegg et al., 2017) although this is not always the case (Hargittai and Jennrich, 2016). In our sample, a tendency for young men to dismiss surveys may partially explain the deficiency of equal participation among the sexes. Because data were drawn from a balanced population, such a large discrepancy by sex (∼34%) can also indicate refusal to participate by some male subjects.

The likelihood a person will respond to a survey may depend upon factors related to the subject matter of the survey. For example, participation in surveys about health is correlated with individuals’ past medical expenditures and their physical health status (Etter and Perneger, 1997; Ness et al., 2010). Lifestyle and cultural background including immigration status can also play an important role in survey response (Goldberg et al., 2001; Rueegg et al., 2017). In ethics research, the Hawthorne effect and manifestations of social desirability response bias have been well-documented, especially when socially sensitive items are present (Randall et al., 1993; Mortel, 2008; Chen et al., 2015).

Given the politically and socially charged nature of a survey that deals with topics of gender roles and discrimination, this and similar studies are not immune to some participants responding unfairly and others opting out completely (Sedgwick, 2013). When accounting for the relative overrepresentation of women and sexual minorities in this study, it is reasonable to assume that the topic of gender discrimination may appear more interesting or relevant to certain groups (i.e., lesbian women) than others (i.e., heterosexual men).

Despite drawing from an evenly distributed pool of panelists which represented a roughly evenly divided target population, relatively few males elected to respond to the survey. The low response rate may indicate less comfort discussing gender-related topics among men. Given that the male demographic was generally less tolerant, it is possible that some men were reluctant to participate in a study that would solicit their negative opinions. It is also possible that women were simply more eager to respond, perhaps because studies on gender are often designed to document discrimination against women. Relevance of the survey to particular groups may have contributed to oversampling.

## Conclusion

There seems to be an association between heteronormative attitudes, personal gender conformity, and tolerance as predicted. Overall, participating students were moderately accepting of gender non-conforming persons and their attitudes were not especially heteronormative. Lesbian and bisexual females were the most accepting of non-conforming persons, while heterosexual males were the least tolerant and held more traditional views about gender expression. LGBT respondents were less conforming and four times as likely to be bullied for non-conformity. Men were generally more conforming as well as more likely than women to be mistreated for gender non-conformity, as male gender roles are often more strictly enforced. The large response disparity among sexes may indicate that men are less comfortable with the topic of gender despite its broad influence.

Despite generally low levels of reported intolerance and heterosexism on average, discrimination persists. Self-reported attitudes influenced by unconscious bias also have a tendency to underestimate the true extent of intolerance (Mortel, 2008). Results of this study may be used in the future to develop interventions aimed at lowering the incidence of discriminatory attitudes and behaviors in society. The apparent association between traditional attitudes, highly conforming behavior, and reduced tolerance will be crucial to inform the design of potential targets for intervention. A baseline understanding of existing attitudes by demographic is also important to focus these approaches on individuals who would most likely benefit. Further research is necessary to better describe and address factors that encourage development of tolerant belief systems and ultimately protect vulnerable populations.

## Ethics Statement

The study was carried out in accordance with the recommendations of the Institutional Review Board at Brigham Young University. Survey respondents were informed of their rights as research participants, as well as risks and benefits of participation, and informed that participation in the survey implied consent. The protocol was approved by the Institutional Review Board at Brigham Young University.

## Author Contributions

SD conceived of the idea to explore the presented topic. SD and BM formed the hypotheses, assembled validated measures, established research methods, and collected data after acquiring IRB approval. BM, CJ, and GA conducted statistical analysis and designed relevant tables. SD and BM contributed to the final manuscript. BM supervised the project.

## Conflict of Interest Statement

The authors declare that the research was conducted in the absence of any commercial or financial relationships that could be construed as a potential conflict of interest.
